# How did that interaction make you feel? The relationship between quality of everyday social experiences and emotion in people with and without schizophrenia

**DOI:** 10.1371/journal.pone.0223003

**Published:** 2019-09-30

**Authors:** Jasmine Mote, David E. Gard, Rachel Gonzalez, Daniel Fulford

**Affiliations:** 1 Sargent College of Health & Rehabilitation Sciences, Boston University, Boston, Massachusetts, United States of America; 2 Department of Psychology, San Francisco State University, San Francisco, California, United States of America; 3 Department of Psychological & Brain Sciences, Boston University, Boston, Massachusetts, United States of America; Harvard University, UNITED STATES

## Abstract

People with schizophrenia report positive emotion during social interactions in ecological momentary assessment (EMA) studies; however, few of these studies examine the qualities of social interactions (e.g., intimacy) that may affect emotion experience. In the current EMA study, people with (*n* = 20) and without schizophrenia (*n* = 15) answered questions about the quality of their social interactions, including their emotion experiences. We also explored the relationship between EMA-reported social experiences and trait loneliness, negative symptoms, and social functioning. People with and without schizophrenia did not differ in EMA-reported proportion of time spent with others, extent of involvement during social interactions, intimacy of interactions, or average number of social interactions. Both people with and without schizophrenia reported more positive than negative emotion during social experiences. However, people with schizophrenia reported more loneliness, more severe negative symptoms, and impaired social functioning compared to people without schizophrenia. Further, specific qualities of social interactions (intimacy of interaction, involvement during interaction) were related to happiness during interactions only in people without schizophrenia. These results suggest that while people with and without schizophrenia report similar rates of in-the-moment social emotion experiences, the impact of social interaction quality on emotion may differ between groups.

## Introduction

“And we sit and drink our coffee/Couched in our indifference/Like shells upon the shore/You can hear the ocean roar/And the dangling conversation/And the superficial sighs/Are the borders of our lives” [1].

Social impairment, including difficulties with social skills, cognition, and motivation, is a cardinal feature of schizophrenia (SZ) [2–4]. People with SZ also report social anhedonia, or a lack of pleasure related to social experiences [5,6]. Anhedonia, both social and non-social, is a component of negative symptoms that has been linked to functioning impairments that are stable across the course of the disorder [7]. However, fundamental research in controlled settings shows that people with SZ have intact hedonic experience in the presence of positively-valenced stimuli [8], as well as evidence of intact hedonic experience in the presence of positive social interactions [9,10]. It may be that people with SZ experience social pleasure in-the-moment but underreport these experiences due to method features of clinical and trait-based assessments (e.g., retrospective reporting, aggregation of affective experiences over time). Thus, we still know relatively little about *when* or *in what contexts* people with SZ may (or may not) experience diminished social pleasure.

Ecological momentary assessment (EMA), also known as the experience sampling method, allows participants to report on experiences as they occur in daily life [11]. Participants are signaled multiple times per day over the course of days or weeks to answer brief questions on their current experiences. Using EMA, participants can report on qualities of social experiences during (or immediately following) a given social interaction. The majority of EMA studies have found that people with SZ spend approximately the same amount of time with others compared to healthy controls [12–15]. People with and without SZ also do not appear to differ in the extent of involvement they report in a given social interaction [13,16], but see [17].

A small subset of EMA studies have examined emotion experience in the context of social interactions in SZ. These studies have found that people with SZ report more positive affect when they are with others compared to being alone [12,18,19], and do not differ from healthy controls in the extent of positive affect experienced during social interactions [14,19]. Fewer EMA studies have examined the qualities of social experiences that influence social emotion experience in people with SZ. Granholm and colleagues (2013) found that people with SZ reported more happiness and less sadness in response to interactions they appraised as being worthwhile and successful, as well as interactions where they believed others perceived them as likeable, smart, and interesting [20]. However, to our knowledge, no study to date has examined whether other qualities, such as involvement in a given social interaction or how close one feels to the person they are interacting with, are related to EMA-reported social emotion experiences in people with SZ. Feeling close to others, including friends, is related to better functioning and quality of life in people with SZ in non-EMA studies (e.g., qualitative interviews) [21,22]. Through EMA, we can better understand the relationship between these subjective qualities of relationships and experiences of social pleasure in daily life.

EMA is an ideal method to examine emotion experiences during or immediately following specific types of social interactions (e.g., during enjoyable experiences, during those with close others) as they occur in the daily lives of people with SZ. EMA does not rely as heavily on retrospective reporting as other methods susceptible to recall bias (e.g., self-reports that require participants to report on past social experiences across weeks or months). Further, EMA does not rely as heavily on cognitive abilities or insight needed to summarize past or hypothetical subjective experiences of social interactions as some self-report methods. Additionally, we can disentangle differences in social emotion experiences from other related factors that trait-based assessments may not capture, such as whether people with SZ differ from those without in the opportunity to engage in social experiences, or the difference between reduced social pleasure and heightened negative affect during social interactions.

In the current EMA study, we investigated “the borders of the lives” of people with and without SZ: momentary social emotion experiences and qualities of social interactions. Based on previous EMA studies, we predicted: (1) People with and without SZ would not differ in proportion of time spent with others, extent of involvement during a given interaction, or average number of social interactions in between EMA signals; and (2) people with and without SZ would report more positive (happiness) and less negative (anxiety, sadness) emotion experience when with others compared to when they were alone. Due to the dearth of previous evidence on the relationship between qualities of social interactions and emotion experience in people with SZ, we explored whether certain qualities of social experiences or number of social interactions were associated with positive and negative emotions during social interactions, and whether this differed between people with and without SZ. As an exploratory aim, we examined correlations between EMA-reported social experiences and psychiatric symptoms and social functioning assessments in people with and without SZ.

## Materials and methods

### Participants

Twenty people with schizophrenia (SZ) and 15 people without SZ (healthy controls; HCs) participated (see Table 1 for demographic information). Participants were recruited from the San Francisco Bay Area. Participants with SZ were recruited via clinician referrals and brochures/flyers posted in local clinics. HC participants were recruited via community flyers and public advertisements on websites typically used to recruit for psychological research studies. Exclusion criteria were the following: a history of head trauma, stroke, neurological disease, or loss of consciousness; a current mood episode; substance dependence within the past six months; not fluent in English; or above the age of 70. For the HC group, people with any past or current *Diagnostic and Statistical Manual of Mental Disorders*, *Fourth Edition* (*DSM-IV-TR*) [23] Axis I diagnosis were excluded.

**Table 1:** Demographic and clinical characteristics.

Characteristic	SZ (*n* = 20)	HC (*n* = 15)	*p* value
Mean Age (SD)	53.30 (7.70)	43.33 (14.15)	.02
% Male gender	75%	73%	.91
Mean Years Education(SD)	14.06 (3.02)	15.57 (3.20)	.22
Mean Employment (SD)	29%	80%	.004
% Married/Cohabitating	18%	1%	.35
% White/Caucasian	35%	60%	.16
% Hispanic/Latinx	6%	0%	.34
% Asian/Asian-American	35%	13%	.15
% Black/African-American	18%	20%	.87
% Multiple Ethnicities	6%	7%	.93
UCLA Loneliness Scale	33.36 (9.59)	21.08 (8.36)	.002
CAINS-MAP	1.97 (0.76)	0.62 (0.52)	< .001
QLS-IR	2.33 (1.52)	4.89 (1.29)	< .001

Notes: CAINS-MAP = Clinical Assessment Interview for Negative Symptoms-Motivation and Pleasure subscale; HC = healthy controls, QLS-IR = Quality of Life Scale-Interpersonal Relations subscale; SD = standard deviation, SZ = people with schizophrenia. All participants reported their age and gender. There is missing data for all other demographic information for three participants with SZ. UCLA Loneliness Scale: SZ (*n* = 14), HC (*n* = 12); CAINS-MAP: SZ (*n* = 18), HC (*n* = 15); QLS-IR: SZ (*n* = 16), HC (*n* = 15).

### Clinical measures

Diagnoses were confirmed by trained research assistants using the *Structured Clinical Interview for DSM-IV–Patient Version* (*SCID-P*) [24]. We assessed negative symptoms in participants using the Clinical Assessment Interview for Negative Symptoms-Motivation and Pleasure subscale (CAINS-MAP) [25]. Higher scores on the CAINS-MAP reflect more severe negative symptoms. We assessed social functioning using the Quality of Life Scale-Interpersonal Relations subscale (QLS-IR) [26], where higher scores reflect better social functioning. Finally, we assessed trait loneliness in participants using the 20-item UCLA Loneliness Scale (UCLA-LS) [27], where higher scores reflect more severe loneliness.

### Ecological momentary assessment (EMA)

Using the Ethica Data application, participants completed brief surveys three times per day for seven consecutive days on researcher-provided smartphones. Similar frequencies have been used in prior EMA studies assessing social behavior in people with serious mental illness [20,28]. Surveys were administered a minimum of 90 minutes apart throughout each day at pseudorandom time points within the windows of 10:00AM-1:00PM, 2:00PM-5:00PM, and 5:00–8:00PM. As part of a larger EMA study (other data in preparation for publication), participants were asked a variety of questions related to their current social context (see Table 2 for EMA questions and forced-choice responses). To determine whether a participant was interacting with another person, an initial forced-choice question of whether the participant was alone (alone or alone with someone else around) or with others was asked. When participants reported being with others, they were also asked to report on how well they knew the person they were with (i.e., intimacy of relationship), how much they were interacting with that person, and how much they were enjoying the interaction. “Interactions” were not explicitly defined as either in-person or over the telephone/through other technology. Regardless of whether participants were with others or not, they were also asked to report on how happy, anxious, and sad they felt in that moment. Finally, participants were asked to report on the number of social interactions they experienced since the last EMA signal, regardless of whether they were currently with others or not.

**Table 2:** Description of EMA prompts and response choices.

EMA Prompt	Response Choices
• How [happy/sad/anxious] are you feeling right now? • How much are you interacting (i.e., talking, playing, etc.) with the people around you?^1^ • How much are you enjoying these interactions?^1^	(0) Not at all, (1) Slightly, (2) Somewhat, (3) Moderately, (4) Very much, (5) Extremely
How well do you know this person?^1^	(0) I don’t know the people here, (1) I know these people a little, (2) I am close to these people, (3) These people are family/very close friends/partners
Since the last prompt, how many times did you talk or communicate with someone?	(0) No interaction, (1) 1 interaction, (2) 2 or 3 interactions, (3) 4 or more interactions

^1^ Prompt was only provided if participant responded that they were with others at the time of the prompt.

### Study procedures

Institutional Review Board approval was obtained from San Francisco State University. Potential participants completed a brief phone screening prior to being invited to participate in the study. Once invited to participate, participants met with a trained research assistant and completed informed verbal and written consent. Researchers determined a participant’s capacity to provide consent through asking questions related to the participant’s comprehension of what was being asked of them to participate in the study as well as confirming with the participant that they were able to sign legal documents on their own behalf. Next, participants completed the SCID-P to confirm diagnostic status and provided demographic information. The researcher then provided the participant with a smartphone and introduced them to the Ethica Data application, including the EMA portion of the study. One week later, participants returned the phone to the researcher (either by returning to the research laboratory where the original assessment took place, or during a home visit the researcher made as part of a larger ongoing study). All other clinical assessments (CAINS, QLS-IR, and UCLA-LS) were completed at study termination, when possible. The clinical assessments were not the main focus of this study and were completed for a subset of participants (see Results for *n* sizes for each assessment).

### Analytic procedures

We examined emotions across experiences (i.e., regardless of whether participant reported being with others or not) and only during social experiences (i.e., only when participant reported being with others). Given the nested structure of EMA data (responses nested within participants), we conducted multilevel analyses using Hierarchical Linear Modeling (HLM) Version 7.03 [29]. Based on adherence standards from other EMA studies in SZ [12,30], participants were required to respond to a minimum of 25% of EMA prompts to be included in analysis; all participants met this minimum threshold. Approximately 30% of the observations were missing (735 out of a possible 1,253; no differences in missing data between people with and without SZ, *t* (33) = 0.17, *p* = .87, Cohen’s *d* = 0.06). Parameters were estimated with full-information maximum likelihood. EMA responses were treated as repeated measures (Level 1) that were nested within persons (Level 2). Additionally, time point (of a possible 21 EMA signals over the course of seven days) was included in each model as a Level 1 predictor, although these results are not reported. Group status (SZ vs. HC) was examined as a Level 2 predictor. Separate group analyses were run if group status was marginally significant in any model (*p* > .10). All person-level intraclass correlations (ICC) for each model were > .10, indicating sufficient interdependence of responses to necessitate multilevel analyses. Ninety-five percent confidence intervals (95% CI) are reported for all multilevel results. We used chi-square and independent-samples *t* tests to examine between-group differences in demographic factors and EMA-assessed means of emotion and social experience variables. Effect sizes (φ and Cohen’s *d* for chi-square and *t* tests, respectively) are reported. We computed Pearson correlations separately for the SZ and HC groups. See S1 and S2 Files for this study’s data.

## Results

See Table 1 for demographic information. Data on age and gender for all participants are reported. Three participants with SZ had incomplete demographic information—additional demographic data (e.g., education) were lost when an error occurred in saving the data electronically. People with SZ (*n* = 20, *M* = 53.30, *SD* = 7.70) were significantly older than the HC group (*M* = 43.33, *SD* = 14.15; *t* (33) = 2.47, *p* = .02, *d* = .91). A smaller proportion of people with SZ were employed (5/17) during the study compared to HCs (12/15; chi-square = 8.19, *p* = .004, φ = .51). Preliminary analyses showed that neither age nor employment status were significantly (*p* > .05) related to any of our social emotion outcome variables; thus, they were not included in subsequent analyses. People with SZ (*n* = 20) did not significantly differ from the HC group in ratio of men to women (chi-square = 0.01, *p* = .91, φ = .02). People with SZ (*n* = 17) also did not significantly differ from the HC group in years of education (*t* (29) = 1.26, *p* = .22, *d* = 0.45), ethnic background (chi-squares = 0.01–2.05, *p*s > .15, φs = .02-.25), or marriage/cohabitation status (chi-square = 0.88, *p* = .35, φ = .17).

A subset of participants completed all clinical measures (see Table 1). Eighteen participants with SZ completed the CAINS-MAP; 16 participants with SZ completed the QLS-IR; and 14 people with SZ and 12 HCs completed the UCLA-LS. People with SZ reported significantly more loneliness on the UCLA-LS compared to HCs (*t* (24) = 3.39, *p* = .002, *d* = 1.36). People with SZ also reported more severe negative symptoms on the CAINS-MAP compared to HCs (*t* (31) = 5.84, *p* < .001, *d* = 2.02). Finally, people with SZ reported significantly lower social functioning on the QLS-IR compared to HCs (*t* (29) = 5.02, *p* < .001, *d* = 1.81).

### Group differences in emotions experienced

Two people with SZ reported being alone during 100% of EMA signals; thus, only 18 people with SZ reported on social emotion experiences (i.e., emotions experienced during social interactions). People with and without SZ did not significantly differ in reports of anxiety across experiences (*d* = 0.36), anxiety during social interactions (*d* = 0.28), sadness across experiences (*d* = 0.40), or sadness during social interactions (*d* = 0.06) (see Table 3). There was a trend association that people with SZ reported less happiness across experiences (*M* = 2.62, *SD* = 1.13) compared to the HC group (*M* = 3.23, *SD* = 0.84; *t* (33) = 1.76, *p* = .09, *d* = 0.60). There was also a trend association that people with SZ reported less happiness during social interactions (*M* = 2.63, *SD* = 1.28) compared to the HC group (*M* = 3.29, *SD* = 0.84; *t* (33) = 1.78, *p* = .09, *d* = 0.60). Within each group, participants reported more happiness than either anxiety or sadness across experiences as well as during social interactions (*p*s < .001; *d*s for SZ = 1.18–1.54; *d*s for HC = 1.82–2.72). Further, within each group, participants did not differ in reports of anxiety compared to sadness across experiences (SZ: *p* = .41, *d* = .18; HC: *p* = .37, *d* = 0.24) or during social interactions (SZ: *p* = .98, *d* = .003; HC: *p* = .60, *d* = 0.13).

**Table 3:** EMA emotion and social item mean scores.

Outcome	SZ (*n* = 20)	HC (*n* = 15)	*p* value
Happiness, across experiences	2.62 (1.13)	3.23 (0.84)	.09
Happiness during social interactions^1^	2.63 (1.28)	3.29 (0.84)	.09
Sadness, across experiences	0.84 (0.66)	0.56 (0.75)	.27
Sadness during social interactions^1^	0.89 (0.81)	0.83 (1.07)	.87
Anxiety, across experiences	0.94 (0.72)	0.70 (0.62)	.29
Anxiety during social interactions^1^	0.89 (0.79)	0.69 (0.61)	.43
Number of interactions since last signal	1.71 (0.62)	1.78 (0.50)	.73
% of signals with others	35%	39%	.63
Intimacy of relationship in interaction^1^	1.55 (0.58)	1.27 (0.76)	.25
Involvement in interaction^1^	2.00 (0.96)	2.32 (1.31)	.43
Enjoyment of interaction^1^	2.80 (1.13)	2.95 (0.88)	.69

^1^ People with SZ, *n* = 18.

### Group differences in quantity and quality of social interactions

People with and without SZ did not differ on any EMA-reported outcome related to quality of social experiences, including proportion of time spent interacting with others (*d* = 0.18), intimacy of relationship during interactions (*d* = 0.42), the extent of involvement during interactions (*d* = 0.28), enjoyment of interactions (*d* = 0.15), and the number of interactions since the last EMA signal (*d* = 0.12) (see Table 3). As stated above, two people with SZ reported being alone during 100% of EMA signals. Overall, participants spent approximately 1/3 of their time during EMA signals interacting with others and reported an average of 1–3 interactions in between signals.

### Qualities of social interactions and social emotion experience

#### Intimacy of relationship

Level of intimacy of relationship with the person a participant was interacting with was significantly related to more happiness during social interactions (*b* = 0.39, *t* (31) = 2.85, *p* < .001, 95% CI = 0.20, 0.59). There was a significant group main effect in this relationship (*b* = -0.56, *t* (31) = 4.03, *p* < .001, 95% CI = -0.29, -0.83). This relationship was significant in the HC group (*b* = .41, *t* (14) = 2.92, *p* = .01, 95% CI = 0.14, 0.69), but not in people with SZ (*b* = -0.22, *t* (17) = 1.68, *p* = .11, 95% CI = -0.49, 0.04). Thus, intimacy of relationship was positively associated with happiness during social interactions only in the HC group.

Intimacy of relationship was also significantly related to more sadness during social interactions (*b* = 0.36, *t* (31) = 2.12, *p* = .04, 95% CI = 0.03, 0.69). There was a trend for a group main effect (*b* = -0.34, *t* (31) = 1.84, *p* = .08, 95% CI = -0.70, 0.02). However, when looking within the SZ group (*b* = -0.01, t (17) = 0.09, 95% CI = -0.27, 0.24) and the HC group (*b* = 0.41, *t* (14) = 1.28, 95% CI = -0.22, 1.04), the relationship between intimacy of relationship and sadness during social interactions was nonsignificant (*p*s > .25). Intimacy of relationship was not significantly related to anxiety during social interactions (*b* = -0.03, *t* (31) = 0.33, *p* = .75, 95% CI = -0.20, 0.14), and there was no significant group main effect (*b* = -0.04, *t* (31) = 0.35, *p* = .73, 95% CI = -0.28, 0.20).

In summary, how close a participant felt to the person they were interacting with in a given EMA signal was significantly related to more happiness during social interactions across participants, but this relationship only remained significant in the HC group. Intimacy of a relationship was also related to more sadness during social interactions across groups. However, intimacy of relationship was unrelated to anxiety during social interactions.

#### Involvement in interactions

The extent of involvement in a given social interaction was positively associated with happiness during social interactions (*b* = 0.33, *t* (31) = 5.19, *p* < .001, 95% CI = 0.21, 0.46). There was a trend for a group main effect (*b* = -0.18, *t* (31) = 1.92, *p* = .06, 95% CI = -0.37, 0.003). The extent of involvement in an interaction was significantly related to more happiness during social interactions in the HC group (*b* = .34, *t* (14) = 5.64, *p* < .001, 95% CI = 0.18, 0.49). However, this relationship was not significant in people with SZ (*b* = .14, *t* (17) = 1.76, *p* = .10, 95% CI = -0.02, 0.30), suggesting that involvement in social interactions is related to happiness when with others only for the HC group.

There was a trend association that involvement in a given social interaction was related to more sadness during social interactions (*b* = .10, *t* (31) = 1.88, *p* = .07, 95% CI = -0.01, 0.21). There was no significant group main effect in this relationship (*b* = -0.09, *t* (31) = 1.21, *p* = .24, 95% CI = -0.22, 0.05). Involvement in a given social interaction was significantly related to less anxiety during social interactions (*b* = -0.12, *t* (31) = 2.41, *p* = .02, 95% CI = -0.22, -0.02), and there was a trend for a group main effect (*b* = .12, *t* (31) = 1.85, *p* = .08, 95% CI = -0.01, 0.24). Involvement in social interactions was related to less anxiety during social interactions in the HC group (*b* = -0.13, *t* (14) = 2.18, *p* = .05, 95% CI = -0.25, -0.01), but this relationship was nonsignificant in people with SZ (*b* = -0.002, *t* (17) = 0.05, *p* = .96, 95% CI = -0.10, 0.10). Thus, involvement in social interactions appears to only be related to less anxiety during social interactions for HCs.

In summary, the extent of involvement in social interactions was significantly related to more happiness and less anxiety during social interactions, but these relationships only remained significant in HCs. There was a trend that, across participants, involvement in social interactions was related to more sadness during interactions.

#### Enjoyment during interactions

As one might expect, enjoyment of a given social interaction was significantly related to more happiness during social interactions (*b* = 0.55, *t* (31) = 6.19, *p* < .001, 95% CI = 0.38, 0.73). There was no significant group main effect in this relationship (*b* = 0.01, *t* (31) = 0.16, *p* = .88, 95% CI = -0.14, 0.16).

Enjoyment during social interactions was not significantly related to sadness during social interactions (*b* = 0.09, *t* (31) = 0.91, *p* = .37, 95% CI = -0.11, 0.29). There was no significant group main effect (*b* = -0.06, *t* (31) = 0.30, *p* = .77, 95% CI = -0.43, 0.31). Enjoyment during social interactions was also not significantly related to anxiety during social interactions (*b* = -0.11, *t* (31) = 1.57, *p* = .13, 95% CI = -0.26, 0.03), and there was no significant group main effect in this relationship (*b* = 0.16, *t* (31) = 1.70, *p* = .10, 95% CI = -0.03, 0.34).

In summary, enjoyment of social experiences during EMA signals was related to more happiness during social interactions across groups. Enjoyment of social experiences was not significantly related to either sadness or anxiety during interactions in either group.

#### Number of interactions between EMA signals

The number of interactions in between EMA signals was significantly related to more happiness during social interactions (*b* = .29, *t* (31) = 2.03, *p* = .05, 95% CI = 0.01, 0.57). There was a significant group main effect (*b* = -0.36, *t* (31) = 2.13, *p* = .04, 95% CI = -0.69, -0.03). In the HC group, this relationship was trending towards significance (*b* = .29, *t* (14) = 1.86, *p* = .08, 95% CI = -0.01, 0.60). However, in people with SZ, the number of interactions in between EMA signals was not significantly related to happiness during interactions (*b* = -0.09, *t* (17) = 0.59, *p* = .56, 95% CI = -0.40, 0.21).

The number of interactions in between EMA signals was not related to sadness during social interactions (*b* = 0.15, *t* (31) = 1.01, *p* = .32, 95% CI = -0.14, 0.44). There was no group main effect (*b* = -0.19, *t* (31) = 0.89, *p* = .38, 95% CI = -0.60, 0.22). The number of interactions in between EMA signals was not related to anxiety during social interactions (*b* = -0.12, *t* (31) = 1.13, *p* = .27, 95% CI = -0.34, 0.09), and there was no group main effect in this relationship (*b* = 0.13, *t* (31) = 0.95, *p* = .35, 95% CI = -0.14, 0.41).

In summary, the number of social interactions in between EMA signals was significantly related to more happiness during interactions, but this relationship was only trending towards significance in the HC group and was not significant in people with SZ. The number of interactions in between EMA signals was not significantly associated with sadness or anxiety during interactions in either group.

### Correlations with clinical measures

Within each group, we examined correlations between clinical and trait measures (UCLA-LS, CAINS, QLS-IR) with social emotion experiences and EMA-reported qualities of social interactions (intimacy of relationship, involvement in interaction, enjoyment of interaction, number of interactions in between EMA signals) (see Table 4). In people with SZ, the UCLA-LS was significantly negatively correlated with happiness when with others and intimacy of relationship during interactions. In other words, higher trait loneliness was related to less happiness during social interactions and interactions that involved less intimacy for people with SZ. The QLS-IR trended towards a significant positive correlation with happiness during social interactions and involvement in interactions, suggesting trends that better social functioning was related to more happiness when with others and more involvement during social interactions for people with SZ.

**Table 4:** Correlations between clinical and social functioning assessments with EMA outcomes.

		Happiness during social interactions	Sadness during social interactions	Anxiety during social interactions	Intimacy of relationship during interaction	Involvement in interaction	Enjoyment of interaction	Number of interactions since last EMA signal
UCLA-LS	SZ	**r = -.65** p = .02* n = 12	r = .36 p = .25 n = 12	r = .32 p = .31 n = 12	**r = -.71** p = .009* n = 12	r = -.28 p = .39 n = 12	r = -.35 p = .27 n = 12	r = -.07 p = .83 n = 14
	HC	r = .48 p = .11 n = 12	r = .17 p = .61 n = 12	r = -.14 p = .67 n = 12	r = .28 p = .38 n = 12	r = .01 p = .98 n = 12	r = .44 p = .15 n = 12	r = .24 p = .45 n = 12
CAINS- MAP	SZ	r = .36 p = .17 n = 16	r = .08 p = .78 n = 16	r = .14 p = .60 n = 16	r = -.28 p = .30 n = 16	r = -.40 p = .13 n = 16	r = -.32 p = .23 n = 16	r = .15 p = .56 n = 18
	HC	**r = -.52** **p = .05** **n = 15**	r = .33 p = .23 n = 15	**r = .54** p = .04* n = 15	**r = -.66** p = .008* n = 15	r = -.15 p = .61 n = 15	**r = -.70** p = .004** n = 15	**r = -.73** p = .002** n = 15
QLS-IR	SZ	**r = .47** **p = .09** **n = 14**	r = -.33 p = .25 n = 14	r = -.08 p = .79 n = 14	r = .46 p = .10 n = 14	**r = .54** **p = .05** **n = 14**	r = .36 p = .20 n = 14	r = -.15 p = .59 n = 16
	HC	r = .28 p = .32 n = 15	r = -.14 p = .61 n = 15	**r = -.56** p = .03* n = 15	**r = .70** p = .004** n = 15	r = .27 p = .33 n = 15	**r = .65** p = .008* n = 15	**r = .69** p = .004** n = 15

Notes: CAINS-MAP = Clinical Assessment Interview for Negative Symptoms-Motivation and Pleasure subscale; QLS-IR: Quality of Life Scale-Interpersonal Relations subscale; UCLA-LS: UCLA Loneliness Scale.

* *p* < .05

**p < .005

In the HC group, the CAINS-MAP was negatively correlated with EMA reports of enjoying a given interaction, intimacy of interactions, and number of interactions between signals. Thus, in HCs, negative symptoms were related to less enjoyment of social experiences, lower intimacy during interactions, and a lower frequency of interactions in between EMA signals. The CAINS-MAP was also positively correlated with anxiety when with others and a trend association with less happiness when with others, suggesting that negative symptoms are related to more anxiety and less happiness when with others in HCs. The QLS-IR was positively correlated with enjoying interactions, intimacy of interactions, and number of interactions in between EMA signals, while it was negatively correlated with anxiety when with others. Thus, better social functioning was related to more enjoyment of social experiences, more intimacy during interactions, higher frequency of interactions in between EMA signals, and less anxiety during social interactions for people without SZ.

## Discussion

We assessed the connections between EMA-reported emotion in the context of daily social interactions and qualities of these interactions in people with and without SZ. Overall, groups did not differ in various qualities of social experiences assessed through EMA, including the proportion of time spent alone, as well as the intimacy, involvement, enjoyment, and average number of social interactions. People with and without SZ reported more happiness than sadness or anxiety when interacting with others, replicating other studies that show no difference between these groups in social emotion experiences [12,14,19]. However, the relationships between qualities of social experiences and emotion differed between groups: the connections between social pleasure (i.e., happiness when with others) and intimacy and involvement during social interactions was only present in those without SZ.

To our knowledge, this is the first EMA study to examine the relationship between intimacy of and involvement in social interactions and social emotion experiences in people with and without SZ. Our results suggest that qualities related to intimacy and involvement may be less related to social pleasure in people with SZ compared to others. While intimacy and involvement might be thought to reflect interpersonal closeness, they do not necessarily indicate a positive or pleasant interaction. Indeed, our findings suggest that higher intimacy of interactions was associated with more sadness across groups. This finding may reflect the types of conversations that occur with loved ones (e.g., those high in emotional difficulty), which are more likely to result in negative emotions, like sadness or anger. Previous studies have found that people with SZ experience heightened interpersonal conflict compared to others, particularly with close others such as family members [31]. While speculative, it may be that people with SZ in our sample experienced more interpersonal conflict than HCs during their social interactions, particularly in interactions higher in intimacy or involvement, resulting in a lack of connection between quality of social experience and social happiness. Differences in rates of interpersonal conflict may also partially explain why involvement in social interactions was related to less anxiety during interactions, but only for HCs. Future EMA studies should incorporate assessments of interpersonal conflict to better understand associations between intimacy and social emotion experience in people with SZ. While people with and without SZ in our study did not differ in reported experiences of anxiety or sadness during social experiences, inclusion of a broader array of negative emotion words may better capture interpersonal stress (e.g., anger, shame). Further, to better understand the dynamic nature of social emotion experiences within a specific relationship, future work could incorporate EMA reports from a caretaker or close other in study design [32].

None of the qualities of daily social interactions we studied were significantly related to happiness when with others in people with SZ. It may be that beliefs regarding the interactions themselves, rather than qualities such as involvement and intimacy, are more directly related to social emotion experience in people with SZ. One previous EMA study found that positive interaction appraisals (e.g., whether the interaction was worthwhile, whether one was perceived as likeable during the interaction) were related to more happiness during interactions in people with SZ [20]. People with SZ report defeatist performance beliefs (e.g., “If I fail partly, it is as bad as being a complete failure”) more frequently than people without SZ, and these beliefs are related to negative symptoms and poorer social functioning [33,34]. Thus, factors such as whether one perceived the interaction as successful or that one was perceived as likeable may be more tied to momentary experiences of social pleasure in people with SZ than the qualities we measured. Additionally, other qualities of interactions may be more closely related to social pleasure in people with SZ than the factors we assessed, such as whether the participant initiated the interaction or not, the reason for the interaction (e.g., buying something at the supermarket, spending time with a friend), and/or the content of the conversation that took place. Future studies should continue to examine these factors and their relationship with social emotion experience in people with and without SZ.

There were no group differences in any EMA-reported indicators of social interaction quality, suggesting that people with SZ did not experience social impairment in daily life (at least in regard to what we measured). However, participants with SZ reported more severe negative symptoms, higher rates of loneliness, and lower social functioning on clinical and trait-based assessments compared to people without SZ, suggesting that they experience *some* form of social impairment. It may be that the qualities of social experience we assessed through EMA (intimacy, involvement, time spent with others, number of interactions) do not directly contribute to overall social functioning in adults with SZ. In our sample, there were few relationships between clinical assessments and EMA social outcomes. Social functioning (QLS-IR) was positively correlated with involvement in social interactions, while trait reports of loneliness were related to less happiness when with others and less intimacy in interactions for people with SZ. Thus, only dispositional loneliness was related to reduced momentary experiences of social pleasure. Overall, people with SZ report more loneliness than the general population [35,36]. It may be that the lonelier a person with SZ is, the less happy they are and the less connected they feel with others. Alternatively, the more a person with SZ interacts with people they are not happy around or feel close to, the lonelier they may feel. Future studies should include a momentary assessment of loneliness to help understand the relationships between loneliness and other aspects of social experiences in the daily lives of people with SZ.

While we did not include a trait measure of social anhedonia, we assessed motivation and pleasure from the past week (CAINS-MAP) and did not find a significant relationship between this assessment and any EMA-assessed social outcome, including emotion experience, in people with SZ. This differs from one other study that found that more severe avolition was related to less positive affect during “unstructured,” non-goal-directed social activities (e.g., at parties or at the movie/theater versus at work or school) in people with SZ [14]. We did not assess specific types of social activities in this same manner. It may be that impairments in motivated behavior are related to social emotion experience during specific social contexts in people with SZ, such as within contexts that may require more motivation to engage/maintain. Future studies should assess various types of social activities and whether pleasure and motivation differ in people with SZ based on type of activity.

Taken together, our findings suggest that “gold-standard” clinical assessments of social functioning and negative symptoms may not be closely tied to social impairment in daily life in people with SZ. An important next step is to carefully examine *what* these assessments are capturing related to social impairment in SZ and *why* these differences exist. Our results suggest that there may be a disconnect between consummatory (in-the-moment) experiences of social pleasure with clinical or trait-based assessments, similar to findings from laboratory-based studies of emotion experience showing intact hedonic responding in people with SZ [8]. While speculative, it may be that, similar to theories regarding differences in trait versus lab-based assessments of anhedonia in SZ, people with SZ have beliefs regarding their capacity to experience social pleasure that vary from their actual experience [37]. In other words, people with SZ may hold generalized beliefs that social experiences are not enjoyable (reflected on trait-based measures), yet experience just as much pleasure during interactions as HCs (reflected in EMA reports). Future studies should continue to examine the coherence between trait and state-based assessments of social experience in SZ to further elucidate when and why this disconnect occurs.

One limitation of our study is that we did not ask participants to differentiate between technology-assisted (e.g., social media use) and other types of social experiences. Recent research in the general population suggests that social media use can be related to increased depression and loneliness, depending on duration of use and other factors [38–40]. A recent review suggests that when people with psychosis have access to and utilize mobile technology and social media, usage rates do not differ from the general population and use is not related to symptoms [41]. However, questions remain as to whether people with SZ have equitable access to smartphones and other devices that facilitate social media use [42], and whether factors related to social media use and negative outcomes are the same in people with SZ as in the general population. Future studies that use smartphones (like ours) can assess both subjective (EMA) and objective (time spent on social media applications, number of text messages sent/received) indicators of social experience to answer these and other questions related to emotion experience and technology-assisted social interactions in people with SZ.

Several other limitations of the current study are worth noting. Sample sizes were small, particularly in examining correlations between clinical assessments and EMA reports of social experiences. These correlational analyses should be considered preliminary and await replication in future studies. It is worth noting that few existing studies have examined coherence between clinical assessments and EMA reports of social experience in SZ. Doing so, even with our smaller sample, provides groundwork for future examination of the ecological utility of such assessments in understanding the everyday social lives of people with SZ. Additionally, we only examined one positive emotion term (happiness) to assess social pleasure. Future studies should include a variety of emotion terms, positive and negative, to capture a more granular assessment of social emotion experience. For example, the “social-conscious” emotions (embarrassment, shame, guilt, pride) may be more salient during social experiences and may be more related to qualities of interactions than emotions that are not specific to social contexts [43]. Further, we utilized clinical assessments (CAINS-MAP, QLS-IR) in both people with and without SZ that were designed to assess social functioning, motivation, and pleasure in SZ specifically. These assessments may be less suitable for evaluating social and motivational constructs in populations that are in the lower range of severity compared to people with SZ. As such, the relationships between these assessments and EMA outcomes in HCs should be interpreted with caution [44]. Additionally, while our frequency of EMA surveys (three times per day over seven days) was similar to other studies, it is still less frequent than the majority of EMA studies that focus on understanding affective experiences and other more temporally dense constructs; thus, a more granular EMA method may have captured nuances that our design did not. Finally, we did not include an independent assessment of trait social anhedonia in our study, which could have helped to clarify the relationship between in-the-moment social pleasure and trait-based assessments.

## Conclusions

People with and without SZ did not differ in overall qualities of social interactions or social emotion experiences in daily life. The relationship between social interaction qualities and emotion experience, however, did differ between groups: intimacy and involvement during social experiences were related to more happiness during interactions, but only for HCs. These results suggest that other factors related to social experiences may influence in-the-moment experiences of social pleasure in people with SZ. Future studies should assess other aspects of social experiences, such as interpersonal conflict, appraisals of experiences, momentary experiences of loneliness, technology-assisted social interactions, and other emotions felt during interactions, to continue to explore what contributes to social pleasure (and displeasure) in people with SZ. In addition, further examination of the coherence between trait and clinical assessments of social functioning and EMA reports of social interactions and emotion experience could speak to the utility of these assessments in evaluating different facets of social experience in SZ.

## Supporting information

S1 File Data. Ecological momentary assessment data.
(CSV)

S2 File Data. Demographics and mean outcomes.
(CSV)
